# Expression of G protein-coupled receptor GPR19 in normal and neoplastic human tissues

**DOI:** 10.1038/s41598-023-46395-3

**Published:** 2023-11-03

**Authors:** Lorena Gerlach, Anna-Sophia Liselott Beyer, Daniel Kaemmerer, Jörg Sänger, Katja Evert, Stefan Schulz, Amelie Lupp

**Affiliations:** 1 Institute of Pharmacology and Toxicology, Jena University Hospital, Jena, Germany; 2 Department of General and Visceral Surgery, Zentralklinik Bad Berka, Bad Berka, Germany; 3 Laboratory of Pathology and Cytology Bad Berka, Bad Berka, Germany; 4 Department of Pathology, University of Regensburg, Regensburg, Germany; 5 Institute of Pathology, University Medicine of Greifswald, Greifswald, Germany; 6 Institute of Pharmacology and Toxicology, Jena University Hospital, Friedrich Schiller University Jena, Drackendorfer Str. 1, 07747 Jena, Germany

**Keywords:** Biomarkers, Molecular medicine, Oncology, Cancer

## Abstract

Little is known about the expression of the orphan G protein-coupled receptor GPR19 at the protein level. Therefore, we developed a rabbit antibody, targeting human GPR19. After verification of the antibody specificity using GPR19-expressing cell lines and a GPR19-specific siRNA, the antibody was used for immunohistochemical staining of a variety of formalin-fixed, paraffin-embedded normal and neoplastic human tissue samples. In normal tissues, GPR19 expression was detected in a distinct cell population within the cortex, in single cells of the pancreatic islets, in intestinal ganglia, gastric chief cells, and in endocrine cells of the bronchial tract, the gastrointestinal tract, and the prostate. Among the 30 different tumour entities investigated, strong GPR19 expression was found in adenocarcinomas, typical and atypical carcinoids of the lung, and small cell lung cancer. To a lesser extent, the receptor was also present in large cell neuroendocrine carcinomas of the lung, medullary thyroid carcinomas, parathyroid adenomas, pheochromocytomas, and a subpopulation of pancreatic neuroendocrine neoplasms. In lung tumours, a negative correlation with the expression of the proliferation marker Ki-67 and a positive interrelationship with patient survival was observed. Overall, our results indicate that in adenocarcinomas and neuroendocrine tumours of the lung GPR19 may serve as a suitable diagnostic or therapeutic target.

## Introduction

With more than 1000 members, G protein-coupled receptors (GPCRs) represent one of the largest human protein superfamilies. GPCRs are essential for many physiological processes, such as regulation of the neuronal, respiratory, cardiovascular, immune, and endocrine systems, as well as for processing light, smell, and taste stimuli^1,2^. They have been implicated in a wide variety of diseases, such as obesity, type 2 diabetes, hypertension, heart failure, depression, schizophrenia, Parkinson’s and Alzheimer’s disease as well as cancer. Therefore, GPCRs represent important drug targets. About 30–40% of all currently prescribed drugs act on GPCRs, and GPCRs are still important targets in the search for new pharmacological treatments^1,2^. Despite extensive research on GPCRs over the last decades, the endogenous ligands of more than 140 receptors are unknown. These so-called orphan receptors, which include the G protein-coupled receptor 19 (GPR19), are of particular interest to drug research^3,4^.

GPR19 was first discovered in 1996 during the screening of a human-expressed sequence tag database^5^. GPR19 mRNA is highly expressed in diverse brain regions, including the olfactory bulb, cortex, hippocampus, hypothalamus, and cerebellum, as well as the mouse, rat, and human pituitary^5–7^. Hence, it has been hypothesized that GPR19 may be involved in feeding, reproductive, defensive, and homeostatic behaviours, as well as learning and memory^7^. There is a high level of GPR19 mRNA in the embryonal mouse brain with a gradual decrease in signal intensity during development, suggesting a role in the early development of the nervous system^7^. It was shown recently that GPR19 mRNA expression in the suprachiasmatic nucleus, the locus of the master circadian clock in the brain, fluctuates in a circadian manner. GPR19 is also involved in the determination of the circadian period length and entrainment to the clock. GPR19-knockout mice exhibit a prolonged circadian period length, delayed initiation of daily locomotor activity, and a reduced capacity for light-induced phase delays but not phase advances. These effects are accompanied by altered circadian clock gene expression in the suprachiasmatic nucleus^8^.

GPR19 mRNA expression is also found in some peripheral organs such as the heart, spleen, liver, kidney, testis, and ovary of mice and rats, albeit at much lower levels than in the brain^5–7^. Apart from its physiological functions, GPR19 may play a role in the development and progression of certain cancers, because GPR19 increases cell proliferation and decreases apoptosis^9^. Based on gene microarray analysis and other molecular techniques, *GPR19* was identified among a group of genes with higher expression in metastatic melanoma samples compared with primary non-metastatic cutaneous cancers^10^. *GPR19* was also found in a set of genes with high expression in stem cell-like glioblastoma cells^11^. Further, GPR19 mRNA is frequently overexpressed in small cell lung cancer (SCLC) and in SCLC cell lines, where it leads to acceleration of the cell cycle^12^. In breast cancers, GPR19 mRNA is elevated, and in GPR19-overexpressing breast cancer cell lines, the receptor drives mesenchymal-like cells to adopt an epithelial-like phenotype^13^.

The peptide hormone adropin has been proposed as an endogenous ligand for GPR19^13,14^. Adropin is produced mainly in the brain and liver but also in the heart, lung, kidney medulla, gastrointestinal tract, muscles, and peripheral blood mononuclear cells. It is additionally present in the circulatory system of animals and humans. Adropin is regulated by core elements of the biological clock and is important for lipid and glucose homeostasis and for maintaining insulin sensitivity. It also regulates water consumption, exerts a protective effect on the cardiovascular system, and improves cognitive function in aged mice^9,15,16^. GPR19 has been proposed to couple to Gαi to inhibit adenylate cyclase^13,17^, and adropin-induced GPR19 stimulation leads to an activation of the MAPK/ERK1/2 pathway^13^.

While there is data on GPR19 mRNA expression, there appears to be no information on the expression of GPR19 at the protein level in normal and neoplastic tissues or cancer cell lines. To close this knowledge gap, in collaboration with Thermo Fisher Scientific (Waltham, MA, USA) we developed a rabbit polyclonal antibody, targeting the carboxyl-terminus of human GPR19. The antibody specificity was first tested using the SCLC cell line OH-1, which expresses GPR19 endogenously, and a GPR19-specific siRNA. We then determined the expression profile for GPR19 for normal human tissues and a wide range of human tumours using a large panel of formalin-fixed, paraffin-embedded, normal and neoplastic human tissue samples. These experiments revealed a strong GPR19 expression almost exclusively in lung tumours. Based on these findings, we expanded the number of lung tumour samples examined and finally evaluated GPR19 expression in a broad panel of different lung tumour entities. The expression results were then correlated with clinical data, such as tumour staging, tumour grading, and overall patient survival.

## Results

### Verification of the specificity of the rabbit anti-human GPR19 antibody

**Figure 1:**
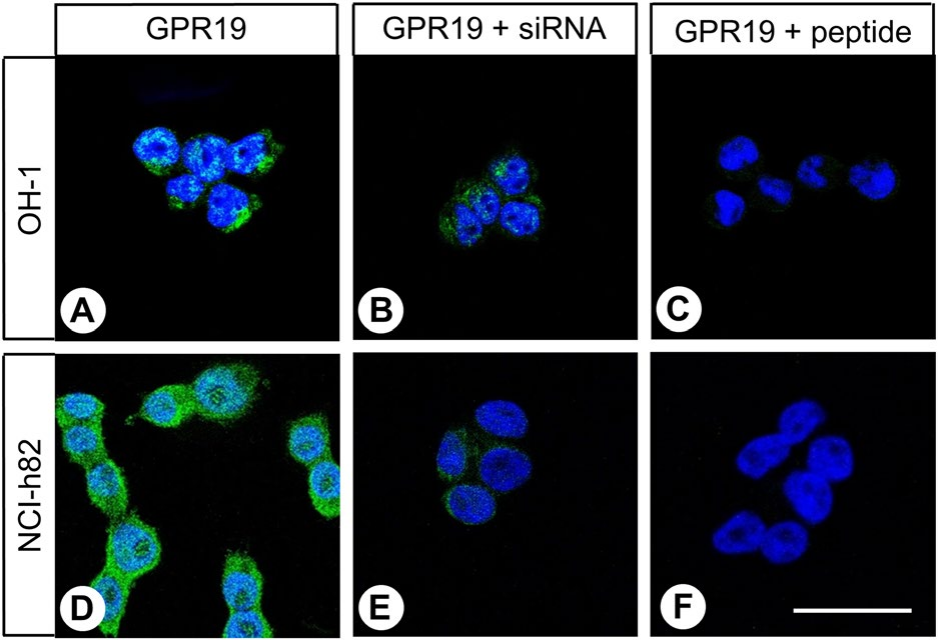
Verification of the specificity of the anti-GPR19 antibody by immunocytochemistry analyses. (**A**) OH-1 cells or (**D**) NCI-h82 cells expressing GPR19 endogenously were fixed and stained with the anti-GPR19 antibody, followed by an Alexa Fluor 488-conjugated anti-rabbit secondary antibody. (**B, E**) For analysis of the specificity of the antibody, GPR19 expression was silenced in OH-1 cells or NCI-h82 cells using a GPR19-specific siRNA. (**C, F**) For adsorption controls, the anti-GPR19 antibody was preincubated for 2 h with 10 µg/ml of the peptide used for immunisations of the rabbits. Green, GPR19 immunosignal; blue, 4′,6-diamidino-2-phenylindole (DAPI) staining of DNA. Scale bar, 50 µm (**A**–**F**).

We first tested the specificity of the anti-GPR19 antibody by immunocytochemistry in OH-1 and NCI-h82 cells, which express GPR19 endogenously, and we found that the receptor was expressed mainly in the cytoplasm of the cells, that is, in an internalised state (Fig. 1A,D). When *GPR19* expression was silenced using a GPR19-specific siRNA, the immunosignal was distinctly reduced (Fig. 1B,E). By contrast, after transfection of the cells with a scrambled siRNA, no difference to the control was observed (not shown). After preincubating the antibody with the immunising peptide, the immunosignal was completely abolished (Fig. 1C,F).

**Figure 2:**
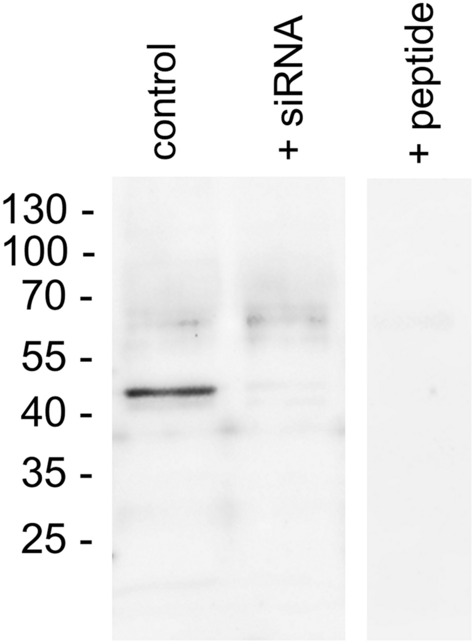
Verification of the specificity of the anti-GPR19 antibody by Western blot analyses. Left lane: Western blot analysis of membrane preparations of OH-1 cells that endogenously express GPR19 (“control”). Middle lane: Western blot analysis of membrane preparations of OH-1 cells after transfection with siRNA targeting GPR19 (“+ siRNA”). Right lane: For adsorption control, the antibody was preincubated for 2 h with 10 µg/ml of the immunising peptide (“+ peptide”). Ladder indicates migration of protein molecular weight markers (kDa). All results are representative of three independent experiments.

The specificity of the anti-GPR19 antibody was further characterised using Western blot analyses in OH-1 cells. When membrane preparations from OH-1 cells were electrophoretically separated and immunoblotted, the antibody recognised a strong band that migrated at approximately 48 kDa (Fig. 2, left lane; “control”). If *GPR19* expression was silenced with a GPR19-specific siRNA, again, a stong reduction in the immunosignal was noted (Fig. 2, middle lane; “+ siRNA”), whereas after transfection of the cells with a scrambled siRNA the immunosignal remained unchanged (not shown). After preincubation of the antibody with the immunising peptide, the immunoreactive band was completely extinguished (Fig. 2, right lane; “+ peptide”).

### Immunohistochemical detection of GPR19 expression in normal human tissues

**Figure 3:**
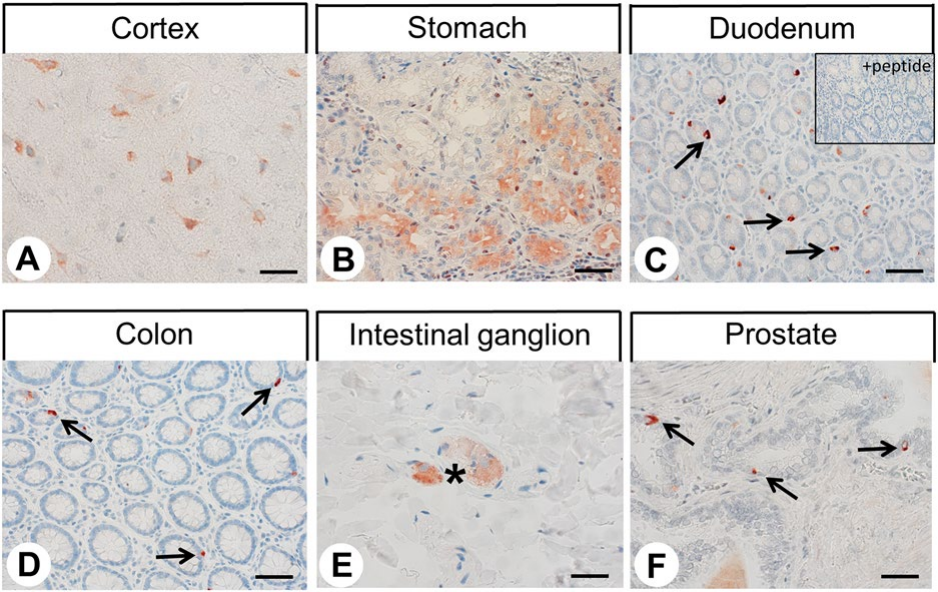
Immunohistochemical detection of GPR19 localisation in normal human tissues. Immunohistochemical staining (red-brown colour) and counterstaining with haematoxylin. Scale bar, 30 µm (**A**,**B**,**E**), 50 µm (**C**,**D**,**F**). Inset in (**C**) adsorption control, in which the anti-GPR19 antibody was preincubated for 2 h with the peptide used to immunise the rabbits (+ peptide). Arrows in (**C**,**D**,**F**), endocrine cells; asterisk in (**E**), intestinal ganglion.

**Figure 4:**
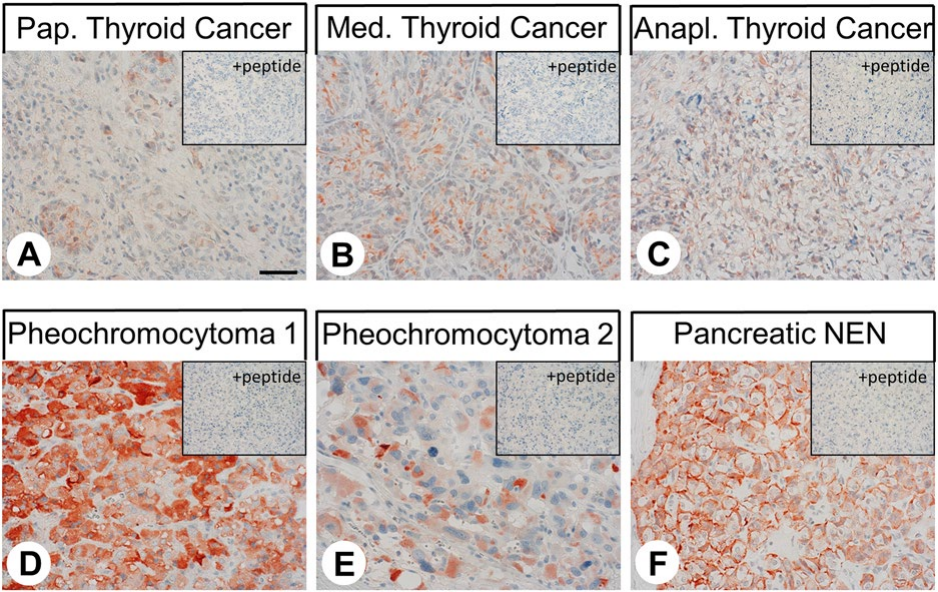
Immunohistochemical detection of GPR19 localisation in human tumour entities. Immunohistochemical staining (red-brown colour) and counterstaining with haematoxylin. Scale bar, 50 µm (**A**–**F**). Insets in (**A**–**F**) represent adsorption controls, in which the anti-GPR19 antibody was preincubated for 2 h with the peptide used to immunise the rabbits (+ peptide).

The rabbit monoclonal anti-GPR19 antibody was then applied to immunohistochemical staining of various normal human tissues. In all cases, both membranous and cytoplasmic staining patterns were observed, with the latter, again, representing internalised receptors. A set of both normal and neoplastic tissues showing positive GPR19 staining was also subjected to immunoadsorption experiments using the immunising peptide, which in all cases led to the complete extinction of the immunosignal (see insets in Figs. 3C, 4A–F). Serial sections of GPR19-positive tissue samples were also stained with the polyclonal rabbit anti-GPR19 antibody PA1-20406 (Thermo Fisher Scientific), which is directed against an amino acid sequence in the third extracellular loop of GPR19, and, thus, to another epitope of the receptor. These additional experiments revealed similar but much less pronounced staining results even at high antibody concentrations as compared to those obtained with the novel anti-GPR19 antibody (Supplemental Fig. 1).

Overall, GPR19 expression was detected in only a few tissues and cell types. Some positive examples are shown in Fig. 3. GPR19 expression was observed in a distinct cell population of the cortex, predominantly in layers II and VI, which, from their morphology, appear to be astrocytes. GPR19 expression was also detected in endocrine cells of the bronchial tract, occasionally in single cells of the pancreatic islets, in intestinal ganglia, and groups of cells located at the base of the gastric glands, most probably representing chief cells. GPR19 expression was additionally found in endocrine cells of the duodenum and (with decreasing frequency) in the jejunum, ileum, and colon, which was confirmed by additional double-labelling fluorescence experiments for chromogranin A (Fig. 4), as well as in endocrine cells of the prostate and in syncytiotrophoblasts of the placenta. Faint staining was noted occasionally in the hepatocytes of the liver. In contrast, no immunostaining was observed in the thyroid gland, alveolar tissue of the lung, heart, spleen, kidneys, lymph nodes, acinar cells of the exocrine pancreas, or in testicular, breast, ovarian, or uterine tissue.

### Immunohistochemical detection of GPR19 expression in various human tumour entities

**Figure 5:**
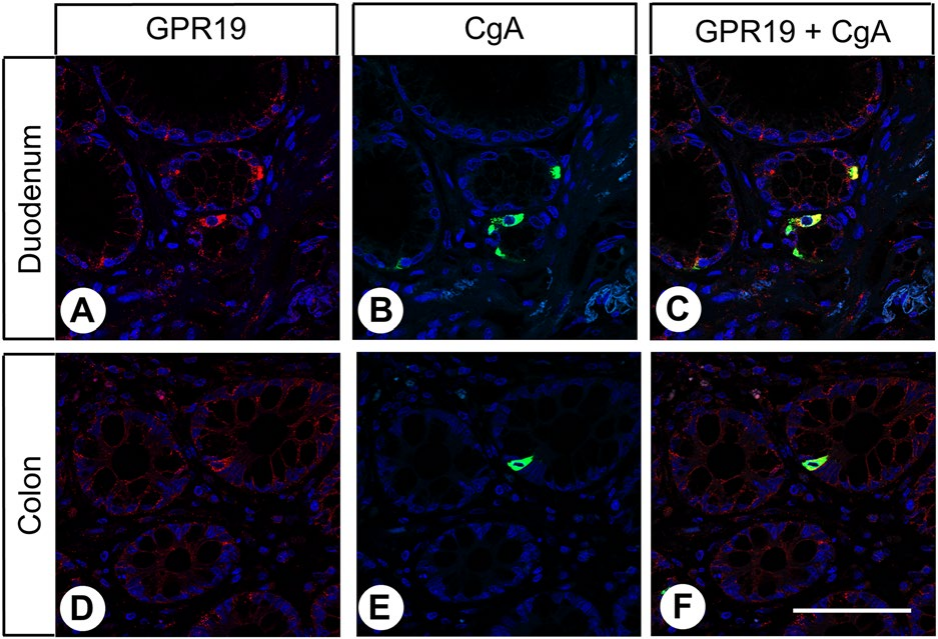
Double-labelling immunohistochemical analysis of GPR19 expression and the expression of chromogranin A (CgA) in human duodenum and colon tissue. Labelling of GPR19 was visualised using Cy3-conjugated goat anti-rabbit antibody (red). Labelling of CgA was visualised using Alexa Fluor 488-conjugated donkey anti-mouse antibody (green). Overlapping expression is represented by orange/yellow colour. Blue colour represents 4′,6-diamidino-2-phenylindole (DAPI)-stained DNA. Scale bar: 100 µm.

The GPR19 expression observed in the different tumour entities is summarised in Table 1. Additionally, representative examples of positively stained tumours are shown in Fig. 5. As observed in normal tissues, both cytoplasmic and membranous staining in tumours were noted. As can be seen from the minimum and maximum Immunoreactivity Score (IRS) values assigned to the individual tumours within the different tumour entities, GPR19 expression displayed substantial inter- but also intra-individual variability. Strong staining was often observed only in small areas of the tumours, with large regions of the samples lacking GPR19, resulting in a low overall score. Higher GPR19 expression, including a greater number of GPR19-positive cases (IRS ≥ 3) and higher IRS values, was particularly prevalent in lung tumours (except for SQC). Besides, noticeable GPR19 expression with average mean IRS values ≥ 3 was observed in only a few tumour entities, including medullary thyroid carcinomas, parathyroid adenomas, and pheochromocytomas. In all other tumour entities, no or only few GPR19-positive tumour samples were observed. However, in single cases for some tumour entities, including papillary thyroid carcinomas, gastrointestinal stromal tumours, pancreatic adenocarcinomas, urinary bladder cancer, and breast cancer, there were medium–high expression levels with IRS values of up to 6 points. Also, among pancreatic neuroendocrine tumours, we found individual tumour samples with IRS values of up to 8.75, corresponding to high expression, although the mean IRS value for this tumour entity was only 2.21.

**Table 1:** Presence of GPR19 in different tumour entities.

Tumour type (total number of cases)	GR19-positive tumours (n)	Immunoreactivity Score (IRS)		
		mean	min	max
Glioblastoma (9)	0	0	0	0
Thyroid carcinoma (38)	17	2.80	0	6
- papillary (10)	5	3.05	2	6
- follicular (10)	1	1.55	0	4.5
**- medullary (9)**	**7**	**5.13**	**2**	**6**
- anaplastic (9)	4	2.17	0	4.5
**Parathyroid adenoma (10)**	**7**	**3.65**	**0**	**6**
**Lung cancer (140)**	**111**	**6.34**	**0**	**12**
** Squamous cell carcinoma (22)**	4	1.44	0	4.5
** Adenocarcinoma (22)**	**20**	**6.13**	**2**	**12**
** Typical carcinoid (21)**	**20**	**7.90**	**2**	**12**
** Atypical Carcinoid (25)**	**24**	**7.52**	**3**	**12**
** Small cell lung cancer (42)**	**38**	**7.91**	**0**	**12**
** Large cell neuroendocrine carcinoma (8)**	**5**	**4.31**	**1.5**	**8**
Gastric adenocarcinoma (9)	1	1.00	0	4
Colon carcinoma (10)	0	0	0	0
Gastrointestinal stromal tumour (10)	4	2.5	0	6
Hepatocellular carcinoma (11)	0	0	0	0
Cholangiocellular carcinoma (10)	3	1.55	0	4
Pancreatic adenocarcinoma (26)	10	2.20	0	8
Pancreatic neuroendocrine tumour (41)	17	2.21	0	8.75
Renal clear cell carcinoma (10)	0	0.30	0	2
Urinary bladder cancer (7)	2	1.79	0	6
**Pheochromocytoma (7)**	**4**	**3.57**	**2**	**5**
Prostate adenocarcinoma (11)	0	0.18	0	2
Testicular cancer (12)	0	0	0	0
Breast carcinoma (10)	2	1.22	0	6
Endometrial cancer (10)	0	0	0	0
Cervical cancer (9)	0	0	0	0
Ovarian cancer (9)	0	0.39	0	2
Lymphoma (10)	1	0.50	0	3
Melanoma (5)	0	1.20	0	2

Tumours with mean IRS values ≥ 3.0 are marked in bold.

### GPR19 expression in lung tumours

#### GPR19 expression pattern

**Figure 6:**
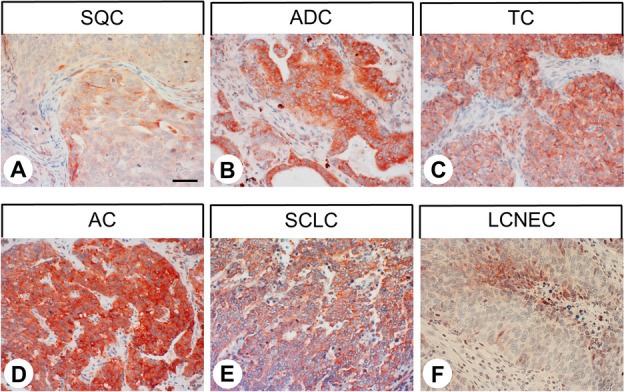
GPR19 expression pattern in different lung tumour entities. Immunohistochemical staining (red-brown colour) and counterstaining with haematoxylin. Scale bar, 50 µm (**A**–**F**).

**Figure 7:**
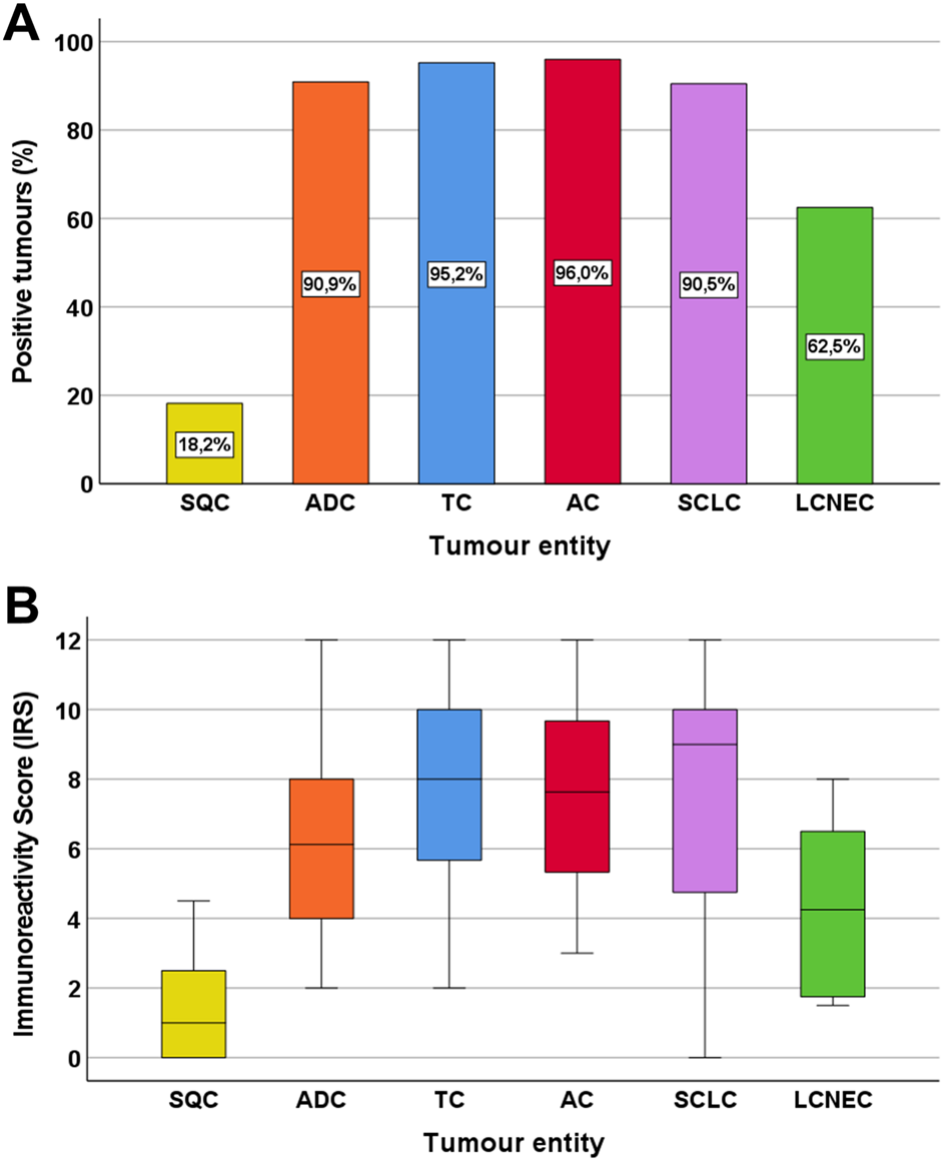
Expression profile of GPR19 in different lung tumour entities. (**A**) Percentage of GPR19-positive cases within the different lung tumour entities. Tumours were only considered positive with Immunoreactivity Score (IRS) values ≥ 3. (**B**) GPR19 expression levels (IRS values) in the different lung tumour entities. Median values, upper and lower quartiles, minimum and maximum values, are shown. SQC, squamous cell carcinomas of the lung; ADC, adenocarcinomas of the lung; TC, typical carcinoids of the lung; AC, atypical carcinoids of the lung; SCLC, small cell lung cancers; LC-NEC, large cell neuroendocrine carcinomas of the lung.

Representative images of GPR19 immunostaining in the different lung tumour entities are shown in Fig. 6. Overall, 79% of the lung tumours tested were GPR19-positive (IRS ≥ 3), with a mean IRS value of 6.34 and a median IRS value of 6.1. Based on the percentage of GPR19-positive tumours (Fig. 7A) and the extent of GPR19 expression (Fig. 7B), GPR19 expression was greatest in typical carcinoids (TC; 95.2% GPR19-positive cases; median IRS value, 8.0), atypical carcinoids (AC; 96.0% GPR19-positive cases; median IRS value, 7.6), and SCLC (90.5% positive cases; median IRS value, 9.0), followed by adenocarcinomas of the lung (ADC; 90.9% GPR19-positive cases; median IRS value, 6.1), large cell neuroendocrine carcinomas of the lung (LCNEC; 62.5% GPR19-positive cases; median IRS value, 4.3), and squamous cell carcinomas of the lung (SQC; 18.2% GPR19-positive cases; median IRS value, 1.0). These differences in GPR19 expression between the different lung tumour entities were statistically significant (Kruskal–Wallis test, p < 0.001; pairwise Mann–Whitney tests, SQC vs. ADC/TC/AC/SCLC, p < 0.001; SQC vs. LCNEC, p = 0.005; ADC vs. SCLC, p = 0.025; LCNEC vs. TC, p = 0.008; LCNEC vs. AC, p = 0.010; LCNEC vs. SCLC, p = 0.007). However, expression levels varied considerably between individual patients, as is evident from the lengths of the boxes and whiskers in Fig. 7B. For example, in SCLC, IRS values from 0 (no expression) to 12 (maximum expression) were observed.

#### Correlations with clinical data

**Figure 8:**
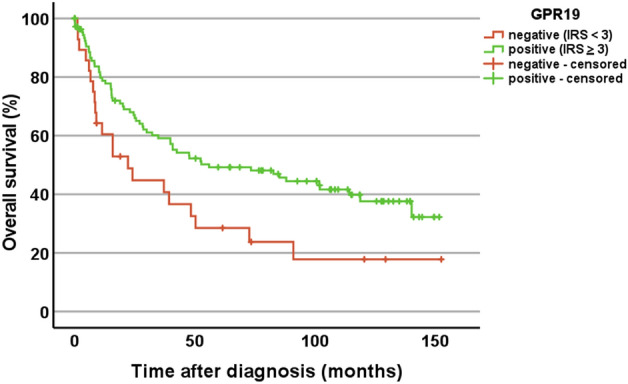
Overall survival of patients with GPR19-positive (IRS ≥ 3) or -negative lung tumours. Log-rank test, p = 0.020. Censored: for the Kaplan–Meier curves, the small vertical ticks mark individual patients whose survival times have been “right censored” because they were still alive at the end of the observation period.

No significant differences in GPR19 expression were noted in dependence of patient age, tumour size, the presence of lymph node metastases, tumour stage, tumour grading, or overall patient survival. There were also no significant differences between primary tumours and metastases or between patients who were still living at the end of the observation period and those who died from tumour-related causes. However, there was a negative correlation between GPR19 expression and the expression of the proliferation marker Ki-67 (Spearman correlation coefficient (rsp) =  − 0.440; p < 0.001), which was corroborated by Kaplan–Meier analyses. When we used either an IRS ≥ 3.0 (the threshold for GPR19-positivity), an IRS ≥ 6.1 (the overall median IRS value of the lung tumours), or an IRS ≥ 5.65 (determined by receiver operating characteristic analysis to represent the optimal threshold for discrimination between groups) as the cut-off for GPR19 positivity, we found that across all lung tumour entities investigated patients with GPR19-positive tumours had significantly better outcomes than those with GPR19-negative tumours (log-rank test: cut-off IRS ≥ 3, p = 0.020 (Fig. 8); cut-off IRS ≥ 6.1, p = 0.047; cut-off IRS ≥ 5.65, p = 0.030). Furthermore, female patients exhibited significantly higher GPR19 expression levels compared with male patients (mean ± SEM: females, 7.63 ± 0.42; males, 5.42 ± 0.41; Mann–Whitney test, p < 0.001). Correspondingly, female patients showed significantly longer survival than male patients (log-rank test, p < 0.001). By contrast, tumours of patients who presented with distant metastases (MTS) at diagnosis had significantly higher IRS values than those of patients without distant MTS (mean ± SEM: without distant MTS, 5.41 ± 0.41; with distant MTS, 7.48 ± 0.71; Mann–Whitney test, p = 0.018).

## Discussion

We developed a GPR19 antibody that could be used for the immunohistochemical staining of formalin-fixed, paraffin-embedded tissues used during routine histopathology analyses. In the present study, we demonstrate that the novel rabbit anti-GPR19 antibody specifically detects its targeted receptor and does not cross-react with other proteins. The antibody revealed cytoplasmic staining of OH-1 and NCI-h82 cells expressing GPR19 endogenously, and the staining was distinctly reduced by treating the cells with a specific *GPR19*-targeting siRNA. In Western blot analyses using membrane preparations from BON-1 cells, the anti-GPR19 antibody detected a band at approximately 48 kDa, which is consistent with the molecular weight reported for the human receptor (Uniprot database; https://www.uniprot.org/uniprotkb/Q15760/entry). *GPR19* knockdown with a GPR19-specific siRNA again led to a strong redction of the immunosignal. Furthermore, the immunosignals obtained in the immunocytochemistry and Western blot analyses as well as in the GPR19-positive tissue samples were completely abolished by preadsorption of the antibody with its immunising peptide.

Finally, when comparing the novel anti-GPR19 antibody with another commercially available polyclonal antibody directed against an amino acid sequence in the third extracellular loop of GPR19, similar staining results were obtained. With the novel anti-GPR19 antibody, however, a more distinct immunosignal and less non-specific background staining were observed, indicating a higher sensitivity and specificity of this antibody. Probably, the C-terminal end of GPR19 represents a better epitope for generating an antibody than the third extracellular loop.

In the present study, strong GPR19 immunostaining was observed in distinct cell populations predominantly located in layers II and VI of the human cortex, which, due to their morphology, most closely correspond to astrocytes. This corroborates in-situ hybridisation data showing GPR19 expression mainly in layers II and VI of the cortex^7^ and other reports on GPR19 mRNA expression in diverse brain regions of rodents and humans, although expression was not assigned to specific cell populations in these studies^5,6^. In peripheral organs, we identified strong GPR19 protein expression in intestinal ganglia, chief cells of the stomach, single cells of some of the pancreatic islets, and neuroendocrine cells of the bronchial and gastrointestinal tracts, and prostate. Therefore, in adults, in addition to its regulatory functions in the central nervous system, GPR19 may also regulate pepsinogen synthesis, gut motility and peptide hormone secretion from neuroendocrine cells throughout the body. Apart from ganglia, chief cells of the stomach and neuroendocrine cells, no noticeable GPR19 expression was seen in the peripheral organs investigated, in contrast to previous reports of GPR19 mRNA expression in the heart, spleen, liver, kidney, testis, and ovary of mice and rats^5–7^. This discrepancy may be due to species differences or to a lack of translation of GPR19 mRNA into protein in these organs.

Among the 30 tumour entities investigated, we identified significant GPR19 expression (mean IRS value ≥ 3) only in lung tumours, especially in those with a neuroendocrine background, such as TC, AC, and SCLC, and in medullary thyroid carcinomas, parathyroid adenomas, and pheochromocytomas, which also belong to the neuroendocrine tumour entities. High GPR19 expression was also found in single pancreatic neuroendocrine neoplasms, probably representing a rare subpopulation of these tumours. It is possible that all these tumours express GPR19 because the cells and tissues of origin have this receptor; however, lung tumours may lose GPR19 expression with increasing malignancy. This is supported by the negative correlation between GPR19 expression and the expression of the proliferation marker Ki-67 and by the better outcomes of patients with GPR19-positive tumours. In other tumour entities, including papillary thyroid carcinomas, gastrointestinal stromal tumours, pancreatic adenocarcinomas, urinary bladder cancer, and breast cancer, we found single tumours, probably representing specific tumour subgroups, with medium–high GPR19 expression. Overall, our findings support the results of previous studies showing elevated GPR19 mRNA expression in metastatic melanomas, glioblastomas, SCLC, and breast cancer^10–13^. For therapeutic or diagnostic relevance, at least medium–high receptor expression in a large number of individual tumours is necessary; this requirement was not met by most of the tumour entities we investigated. However, due to its high expression rate in adenocarcinomas and neuroendocrine tumours of the lung, including TC, AC, and SCLC, with at the same time only very low occurrence in healthy organs, so that only minor side effects are to be expected, GPR19 clearly represents an interesting diagnostic or therapeutic target that should be investigated further.

## Materials and methods

### Antibody

A rabbit polyclonal antibody was produced against the carboxyl-terminus of human GPR19 in collaboration with and obtained from Thermo Fisher Scientific (Waltham, MA, USA; Catalog no.: PA1-151). The peptide used for immunisations of the rabbits was Cys-REAKEKKLAWPINSNPPNTFV, corresponding to residues 395–415 of human GPR19. Because the target sequence is identical, the antibody also detects rat and mouse GPR19.

### Immunocytochemistry

Endogenous GPR19-expressing OH-1 or NCI-h82 cells (DSMZ, Braunschweig, Germany) grown on coverslips overnight were either left untreated or treated with chemically synthesised, double-stranded GPR19 small interfering RNA (siRNA) duplexes (Santa Cruz Biotechnology, Dallas, TX, USA), according to the manufacturer’s instructions. A scrambled siRNA was used as the negative control (Santa Cruz Biotechnology, Dallas, TX, USA). The cells were fixed with 4% paraformaldehyde and 0.2% picric acid in phosphate buffer (pH 6.9) for 20 min at room temperature, washed with phosphate buffer, and incubated with the novel anti-GPR19 antibody (1:100 dilution) overnight at 4 °C, followed by incubation with Alexa Fluor 488-conjugated secondary antibody (Invitrogen, Carlsbad, CA, USA; 1:5,000 dilution) for 2 h at room temperature. Samples were mounted using Fluoromount G (Invitrogen, Carlsbad, CA, USA) and analysed using a Zeiss LSM 510 META laser-scanning confocal microscope (Carl Zeiss, Jena, Germany). For immunostaining controls, the anti-GPR19 antibody was either omitted or adsorbed for 2 h at room temperature with 10 µg/ml of the peptide used for rabbit immunisations.

### Western blot analysis

Endogenous GPR19-expressing OH-1 cells (DSMZ) were seeded onto poly-L-lysine-coated 60-mm dishes and grown to 80% confluence. Cells were either left untreated or treated with chemically synthesised, double-stranded GPR19 siRNA duplexes (Santa Cruz Biotechnology) in accordance with the manufacturer’s instructions. A scrambled siRNA was used as the negative control (Santa Cruz Biotechnology). Subsequently, the cells were lysed in detergent buffer (20 mM 4-(2-hydroxyethyl)-1-piperazineethanesulfonic acid [HEPES, pH 7.4], 150 mM NaCl, 5 mM ethylenediaminetetraacetic acid, 1% Triton X-100, 10% glycerol, 0.1% sodium dodecyl sulphate, 0.2 mM phenylmethylsulfonylfluoride, 10 mg/ml leupeptin, 1 mg/ml pepstatin A, 1 mg/ml aprotinin, and 10 mg/ml bacitracin). GPR19 enrichment was conducted using wheat germ lectin agarose beads (J-OIL MILLS, Inc., Tokyo, Japan), as previously described^18^. Subsequently, the protein content of the samples was determined using the Pierce™ BCA Protein Assay Kit (Thermo Fisher Scientific, Waltham, MA, USA) according to manufacturer’s instructions and the samples (20 µg of protein per lane) were subjected to 7.5% sodium dodecyl sulphate–polyacrylamide gel electrophoresis and immunoblotted onto polyvinylidene fluoride membranes. Blots were incubated with the novel anti-GPR19 antibody (1:100 dilution) overnight at 4 °C, then incubated with peroxidase-conjugated secondary anti-rabbit antibody (1:5000 dilution; Santa Cruz Biotechnology) for 2 h at room temperature and visualised by enhanced chemiluminescence (Amersham, Braunschweig, Germany; Fusion FX7, Vilber Lourmat, Eberhardzell, Germany).

For adsorption controls, the anti-GPR19 antibody was preincubated for 2 h at room temperature with 10 µg/ml of the immunising peptide.

### Immunohistochemical evaluation of GPR19 expression in normal and neoplastic human tissues

#### Tumour specimens

For the initial evaluation of GPR19 expression in different human tumour entities, 304 archived formalin-fixed, paraffin-embedded tumour samples from 304 patients (Table 1; lung tumours originally 10 samples each from ADC, SQC, and SCLC) were obtained from the Department of Pathology of the Ernst-Moritz-Arndt-University (Greifswald, Germany) and the Laboratory of Pathology and Cytology Bad Berka (Bad Berka, Germany). Many of the tumour specimens contained adjacent non-neoplastic tissue, which was also evaluated. Additionally, tumour-free human tissue samples from the cortex, lung, heart, liver, stomach, gut, pancreas, kidney, spleen, lymph nodes, prostate, and testicles (n = 5–10 each) were obtained from the Department of Pathology of the Ernst-Moritz-Arndt-University (Greifswald, Germany) and the Laboratory of Pathology and Cytology Bad Berka (Bad Berka, Germany) for this study.

#### Lung tumour samples

For the subsequent assessment of GPR19 expression in different lung tumours, a total of 328 tumour samples from 140 patients were evaluated. The samples were provided by the Institute of Pathology and Cytology Bad Berka (Bad Berka, Germany) and were surgically removed between 1998 and 2014 at the Department of General and Visceral Surgery, Zentralklinik Bad Berka (Bad Berka, Germany). Clinical data were gathered from patient records. Of the 140 tumours, 22 were SQC, 22 ADC, 21 TC, 25 AC, 42 SCLC, and 8 were LCNEC. Of the tumours, 117 were primary, 16 were metastases, and for 7 samples, no information was available. The clinicopathological characteristics of the patients and tumours are summarised in the Supplemental Table 1. Supplemental Fig. 2 depicts the Kaplan–Meier survival curves for the different lung tumour entities.

All procedures performed in this study involving human participants were in accordance with the 1964 Helsinki declaration and its later amendments. The local ethics committee (Ethikkommission der Landesärztekammer Thüringen) granted permission for this retrospective analysis. Informed consent for the use of tissue samples for scientific purposes was obtained from all study participants when they entered the Theranostic Research Center, Zentralklinik Bad Berka, Bad Berka, Germany, and the Department of General, Visceral and Vascular Surgery, Jena University Hospital, Jena, Germany. All data were analysed anonymously.

#### Immunohistochemistry

From the paraffin blocks, 4-µm sections were prepared and floated onto positively charged slides. Immunostaining was performed using an indirect peroxidase labelling method, as described previously^19^. Briefly, sections were dewaxed and rehydrated via a graded ethanol series, during which endogenous peroxidases were blocked by an additional incubation of the slides in 0.3% H_2_O_2_ in methanol for 45 min. Samples were then microwaved in 10 mM citric acid (pH 6.0) for 16 min at 600 W and incubated with the anti-GPR19 antibody (1:100 dilution) overnight at 4 °C, followed by incubation with biotinylated anti-rabbit IgG and peroxidase-conjugated avidin (Vector ABC “Elite” kit; Vector Laboratories, Burlingame, CA, USA). The binding of the primary antibody was visualised using 3-amino-9-ethylcarbazole in acetate buffer (BioGenex, San Ramon, CA, USA). Sections were counterstained with Mayer’s haematoxylin and mounted in Vectamount™ mounting medium (Vector Laboratories, Burlingame, CA, USA). For immunohistochemical controls, the novel anti-GPR19 antibody was either omitted or adsorbed for 2 h at room temperature with 10 µg/ml of the peptide used for rabbit immunisations. For comparison, a subset of serial sections was additionally incubated with a rabbit polyclonal anti-GPR19 antibody directed against an amino acid sequence in the third extracellular domain of GPR19 (Thermo Fisher Scientific, Catalogue no.: PA1-20406; dilution: 1:50).

For double-labelling fluorescence immunohistochemistry, sections were incubated overnight at 4 °C with the rabbit anti-GPR19 antibody (1:100 dilution) together with a mouse monoclonal anti-chromogranin A (CgA) antibody (1:50 dilution; DAKO, Glostrup, Denmark). The sections were then washed and incubated for 2.5 h in darkness at room temperature with Cy3-conjugated goat anti-rabbit secondary antibody and Alexa Fluor 488-conjugated donkey anti-mouse antibody (1:1000 dilution; Dianova, Hamburg, Germany). Finally, sections were mounted (Fluoromount G, with DAPI; Thermo Fisher Scientific) and evaluated using an LSM 510 META laser scanning confocal microscope (Carl Zeiss, Jena, Germany).

GPR19 single-labelling staining in the tumour samples was scored by the semiquantitative Immunoreactivity Score (IRS), as described by Remmele and Stegner (1987)^20^. The percentage of positive tumour cells in each of five categories (no positive cells, 0; < 10% positive cells, 1; 10%–50% positive cells, 2; 51%–80% positive cells, 3; and > 80% positive cells, 4) was multiplied by the staining intensity quantified in four categories (no staining, 0; mild staining, 1; moderate staining, 2; and strong staining, 3). IRS values ranged from 0–12. When a patient had more than one tumour tissue sample, the arithmetic mean was calculated from the IRS values of the different slides belonging to the same patient. Only tumours with an average IRS ≥ 3 were considered to be GPR19-positive. All immunohistochemically stained samples were evaluated by two independent, blinded investigators (LG, AL). For discrepant scores, final decisions were achieved by consensus.

### Statistical analysis

We used SPSS 28.0.0.0 (IBM, Armonk, NY, USA) for statistical analysis. Because the Kolmogorov–Smirnov test showed that the data were not normally distributed, the Kruskal–Wallis test, Mann–Whitney U test, Chi-square test, Kendall’s τ-b test, and Spearman’s rank correlation were performed. For survival analysis, the Kaplan–Meier method with a log-rank test was used. For all analyses, p values ≤ 0.05 were considered significant.

## Conclusions

We have generated and characterised a novel rabbit anti-human GPR19 antibody that is well-suited for visualising human GPR19 expression in formalin-fixed, paraffin-embedded tissues. This antibody provided for the first time a broad profile of GPR19 protein expression in a wide variety of normal and neoplastic human tissues. In normal tissues, GPR19 was expressed mainly by neuronal and neuroendocrine structures and cells, whereas most tissues were GPR19-negative. Regarding the different tumour types, GPR19 was predominantly expressed in adenocarcinomas of the lung and lung tumours with a neuroendocrine background, and, to a lesser extent, in other neuroendocrine tumour entities such as medullary thyroid carcinomas, parathyroid adenomas, pheochromocytomas, and pancreatic neuroendocrine neoplasms. In adenocarcinomas and neuroendocrine tumours of the lung, GPR19 may serve as a suitable diagnostic or therapeutic target.

### Supplementary Information

Supplementary Information 1.

Supplementary Information 2.

## Data Availability

All data generated during this study are included in this published article. The amino acid sequence of the receptor against which the antibody was raised is publicly available through the Uniprot database (https://www.uniprot.org/uniprotkb/Q15760/entry).

## Abbreviations

AC: Atypical carcinoid
ADC: Adenocarcinoma of the lung
GPR19: G protein-coupled receptor 19
CPCR: G protein-coupled receptor
IRS: Immunoreactivity Score
LCNEC: Large cell neuroendocrine carcinoma of the lung
NEN: Neuroendocrine neoplasm
SCLC: Small cell lung cancer
SQC: Squamous cell carcinoma of the lung
rsp: Spearman correlation coefficient
TC: Typical carcinoid
