# Supplementary material for: Expression of G protein-coupled receptor GPR19 in normal and neoplastic human tissues
Source: Sci Rep. 2023 Nov 3;13:18993. doi: 10.1038/s41598-023-46395-3 (PMC10624815; doi:10.1038/s41598-023-46395-3)
Supplement: Supplementary file 1 — Supplementary Information 1. [file 41598_2023_46395_MOESM1_ESM.pdf]

Supplemental Figure S1

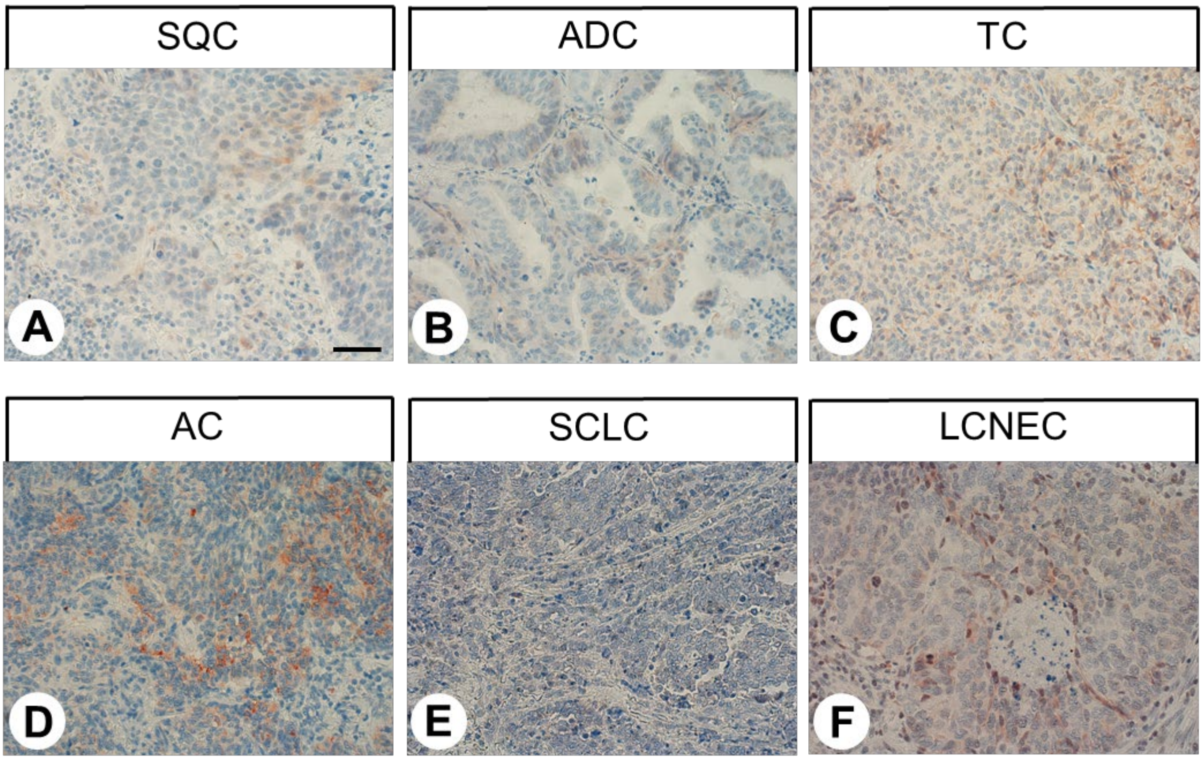

Supplemental Figure S1: Comparative immunohistochemical stainings using the polyclonal rabbit anti-GPR19 antibody PA1-20406 (Thermo Fisher Scientific). Stainings on serial sections of the same samples presented in Figure 6 are shown. Immunohistochemical staining (red-brown colour), counterstaining with haematoxylin. Scale bar: 50 µm (A-F).

Supplemental Table 1

Patient and tumour characteristics. AC, atypical carcinoid; ADC, adenocarcinoma of the lung; LCNEC, large cell neuroendocrine carcinoma of the lung; SCLC, small cell lung cancer; SQC, squamous cell carcinoma of the lung; TC, typical carcinoid; pT, pN, and pM, TNM classification according to the pathology report; unk., unknown; * survival time refers to the patients who died from tumour-related causes.

		SQC	ADC	TC	AC	SCLC	LCNEC	Total
**Total no.**		22	22	21	25	42	8	140
**Sex**	**male**	21	9	5	14	28	5	82
**(number)**	**female**	1	13	16	11	14	3	58
**Age**	**mean**	62.3	65.5	61.7	59.2	58.8	61.6	61.1
**(years)**	**median**	62.8	64.2	63.8	65.1	59.1	60.2	62.2
**Deceased**	**number**	15	15	2	11	36	3	82
**Survival**	**mean**	33.3	28.3	28.3	39.2	27.3	9.7	29.5
**(months)***	**median**	22.2	18.9	28.3	30.0	15.2	9.0	15.8
**pT (n)**	**1**	5	6	11	8	5	2	35
**(number)**	**2**	10	14	4	7	15	3	53
	**3**	6	1	0	1	4	2	14
	**4**	1	1	0	1	12	0	15
	**unk.**	0	0	6	8	6	1	23
**pN (n)**	**0**	9	16	15	11	5	3	59
**(number)**	**1**	13	6	1	5	30	4	59
	**unk.**	0	0	5	9	7	0	22
**pM (n)**	**0**	20	13	14	12	16	4	79
**(number)**	**1**	1	4	1	4	15	1	26
	**unk.**	1	5	6	9	11	3	35

Supplemental Figure S2

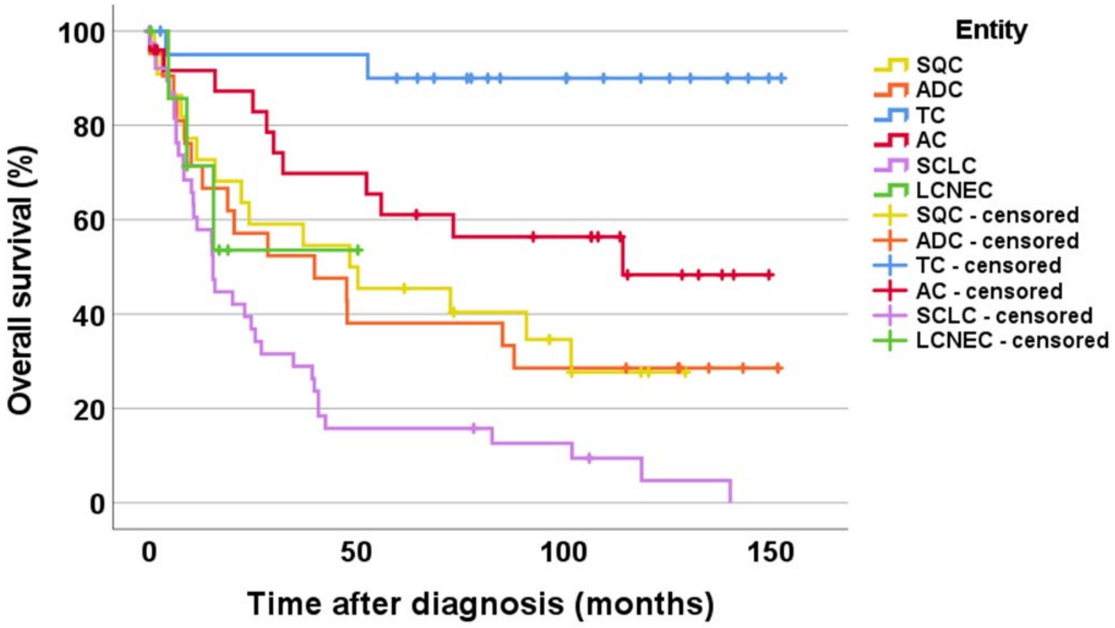

Supplemental Figure S2: Overall survival of patients with squamous cell carcinoma (SQC), adenocarcinoma (ADC), typical carcinoid (TC), atypical carcinoid (AC), small cell lung cancer (SCLC), and large cell neuroendocrine carcinoma of the lung (LCNEC). Log-rank test: $p < 0.001$. Censored: for the Kaplan-Meier curves, the small vertical ticks mark individual patients whose survival times have been “right censored” because they were still alive at the end of the observation period.
