# Supplementary material for: Expression of G protein-coupled receptor GPR19 in normal and neoplastic human tissues
Source: Sci Rep. 2023 Nov 3;13:18993. doi: 10.1038/s41598-023-46395-3 (PMC10624815; doi:10.1038/s41598-023-46395-3)

Other supplemental Material

Original unprocessed versions of the Fusion FX7 images of the membranes used for Figure 2

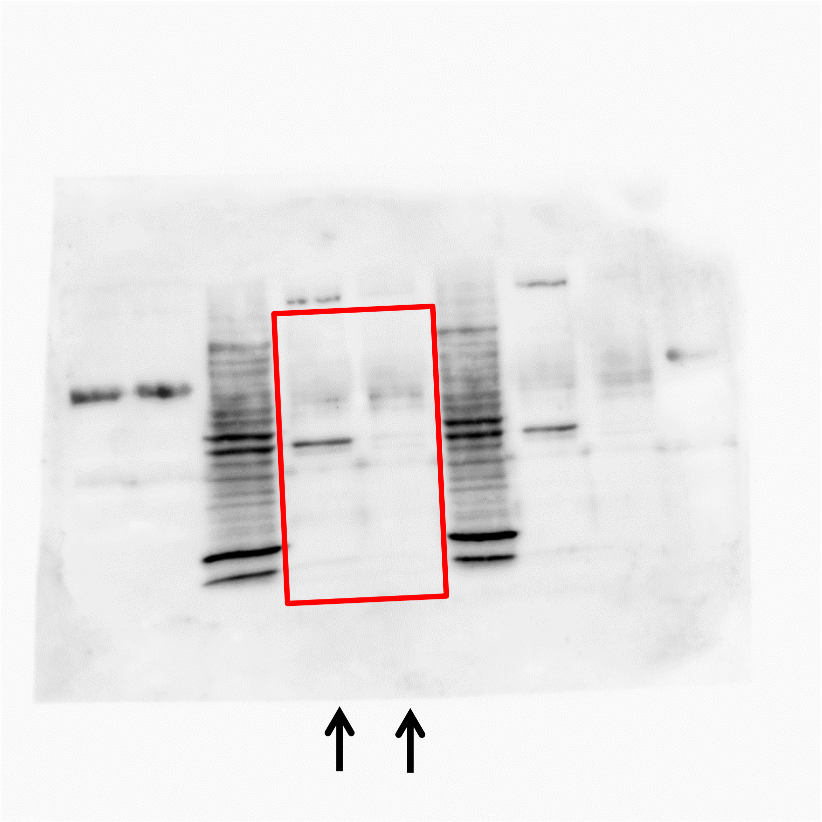

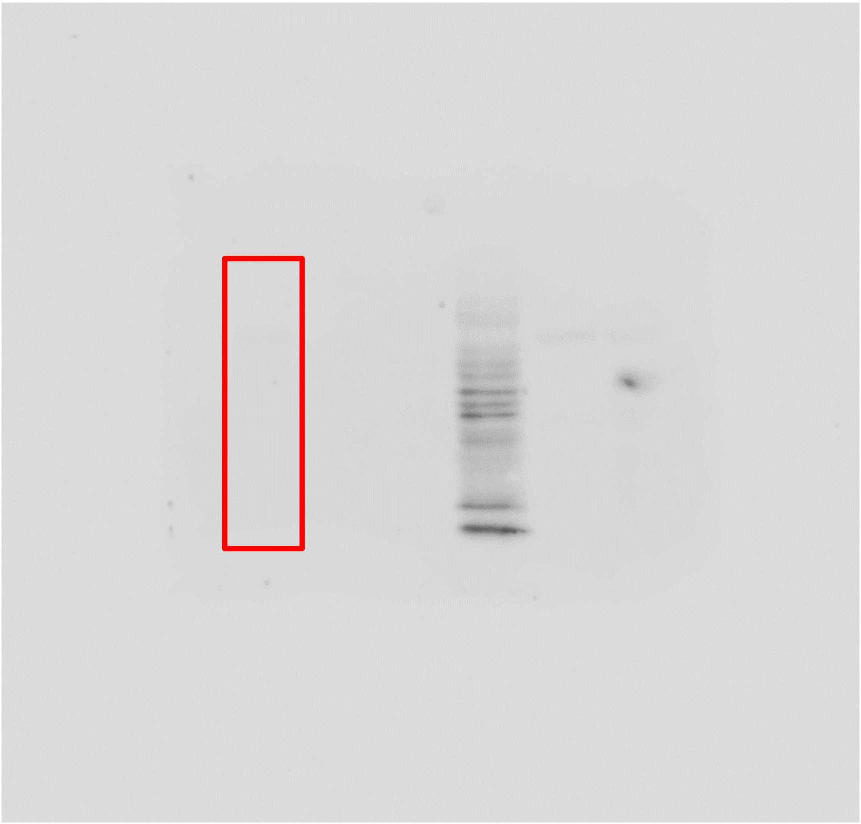

Supplement: Supplementary file 2 — Supplementary Information 2. [file 41598_2023_46395_MOESM2_ESM.pdf]
